# Multilevel exploration of individual- and community-level factors contributing to overweight and obesity among reproductive-aged women: a pooled analysis of Bangladesh Demographic and Health Survey, 2004–2018

**DOI:** 10.1017/S1368980022001124

**Published:** 2022-08

**Authors:** Benojir Ahammed, Md. Alamgir Sarder, Subarna Kundu, Syed Afroz Keramat, Khorshed Alam

**Affiliations:** 1 Statistics Discipline, Science, Engineering and Technology (SET) School, Khulna University, Khulna 9208, Bangladesh; 2 Economics Discipline, Social Science School, Khulna University, Khulna, Bangladesh; 3 School of Business, University of Southern Queensland, Toowoomba, QLD, Australia; 4 Centre for Health Research, University of Southern Queensland, Toowoomba, QLD, Australia

**Keywords:** Overweight, Obesity, Reproductive-aged women, Multilevel analysis, Bangladesh

## Abstract

**Objectives:**

Overweight and obesity have been related to a variety of adverse health outcomes. Understanding the overweight and obesity epidemic in Bangladesh, particularly among reproductive-aged women, is critical for monitoring and designing effective control measures. The purpose of this study was to determine the prevalence of overweight and obesity in reproductive-aged women and to identify the risk factors of overweight and obesity.

**Design:**

A total of 70 651 women were obtained from the five most recent and successive Bangladesh Demographic and Health Surveys (BDHS). The multilevel logistic regression model was used to explore the individual- and community-level factors of overweight and obesity.

**Setting:**

Five most recent nationally representative household surveys across all regions.

**Participants:**

Reproductive-aged (15–49 years) non-pregnant women.

**Results:**

Approximately 35·2 % (95 % CI: 34·9–35·6 %) of women were either overweight or obese in Bangladesh. At the individual- and community-level, higher age (adjusted odds ratio (aOR) = 5·79, 95 % CI: 5·28–6·34), secondary or higher education (aOR = 1·69 [1·60–1·78]), relatively wealthiest households (aOR = 4·41 [4·10–4·74]), electronic media access (aOR = 1·32 [1·26–1·37]) and community high literacy (aOR = 1·10 [1·04–1·15]) of women were significantly positively associated with being overweight or obese. Whereas, rural residents (aOR = 0·79 [0·76–0·82]) from larger-sized households (aOR = 0·80 [0·73–0·87]) and have high community employment (aOR = 0·92 [0·88–0·97]) were negatively associated with the probability of being overweight or obese.

**Conclusion:**

Individual- and community-level factors influenced the overweight and obesity of Bangladeshi reproductive-aged women. Interventions and a comprehensive public health plan aimed at identifying and addressing the growing burden of overweight and obesity should be a top focus.

Globally, the rising prevalence of overweight and obesity is a severe public health concern. Overweight and obesity are the result of imbalance between energy consumption and energy expansion. Excessive body weight is a risk factor for chronic diseases, such as hypertension, diabetics, heart diseases and cancer^(1)^. The number of obese women has increased from 69 million in 1975 to 390 million in 2016^(2)^. This problem severely affects reproductive-aged women because it harms their health and affects the health status of their offspring^(3)^. Women with overweight and obesity have a high risk of infertility and different pregnancy complications, including gestational diabetics, hypertension, haemorrhage and eclampsia^(3,4)^. Understanding the pattern of overweight and obesity and their determinants, especially among reproductive-aged women, is vital to secure the health of current and future generations. Consequently, the prevalence and determinants of overweight and obesity among reproductive-aged women are of particular interest to researchers and policy-makers. The rate of overweight and obesity is rapidly increasing in Bangladesh as a result of rapid urbanisation, economic growth, changes in dietary habits and lifestyle and modernisation^(5)^. The recent Bangladesh Demographic and Health Survey (BDHS) report showed that overweight and obesity among reproductive-aged women have increased from 12 % to 32 % between 2007 and 2017^(6)^. Unlike undernutrition, overweight or obesity is a kind of malnutrition that receive little attention in Bangladesh^(7)^. Because of the rising prevalence of overweight and obesity in Bangladesh, it is necessary to take this issue into consideration while developing health and nutritional policy. Several studies have attempted to discover the associated factors of overweight and obesity in different settings using a single survey, including Bangladesh^(8–10)^. Individual factors associated with overweight and obesity have been discovered previously. However, the risk factors for overweight and obesity at the community level have received scant attention. A recent study looked at the prevalence and risk factors of overweight and obesity among Bangladeshi reproductive-aged women^(11)^. They determined the risk factors for underweight, overweight and obesity using pooled data from 1999 to 2014 and a multinomial logistic regression model. However, community-level factors were not considered in this study. Individual-level factors are deep setters in the community-level factors. Thus, disregarding community-level factors is a flaw of the previous research.

There is strong evidence that community factors, such as wealth status and literacy, have played a role in people being overweight and obese^(7,12)^. As a result, making inferences without considering individual- or community-level factors may result in erroneous information. This study aimed to investigate the individual- and community-level factors responsible for overweight and obesity among reproductive-aged women by conducting a multilevel analysis that considers the clustering and hierarchical structure of different survey years. Multilevel analysis has been utilised in a few research, although the results are outdated. The reason for this is that numerous changes in other socio-economic elements have happened over time, including media campaigns using radio, television and educational status. Bangladesh has raised literacy rate (58·6 % to 72·3 %) and per capita income in recent years^(13)^. Women who live in highly educated societies and have access to the media are more aware of their own health and well-being^(7)^. The reason for this is that the public has access to nutritional information through the media^(14)^. As a result, the literacy rate of women in the community, wealth position, media access and employment may all have an impact on nutritional status and individual-level variables. Using the most recent nationally representative data from Bangladesh, this study examined the patterns and prevalence of overweight and obesity among reproductive-aged women and evaluated its association with individual- and community-level factors.

## Methods

### Data and sampling design

This study analysed data from five consecutive surveys undertaken by the BDHS in 2004, 2007, 2011, 2014 and 2017–2018. The Bangladesh Ministry of Health and Family Welfare and the National Institute of Population Research and Training conducted the survey. All BDHS employed a two-stage stratified cluster sampling procedure for selecting the sample. In the first stage, enumeration areas were randomly selected according to the probability proportional to the size of each enumeration area. In the second stage, the average number of households per enumeration area was chosen for each division to provide reliable sample estimates for the whole country. The surveys included 11 440, 10 996, 17 842, 17 863 and 20 250 married reproductive-aged women from five consecutive cross-sectional household surveys. Missing values on the outcome and explanatory variables and women who were pregnant or had given birth within the previous 2 months of survey visits were excluded. A total of 70 651 women were evaluated for the subsample analyses after the exclusion criteria were met. The detailed information on the sampling techniques, survey design, survey instruments, measuring system, data collections and quality control has been previously described^(6,15–18)^.

## Study variables

### Outcome variables

Overweight and obesity status was considered as the outcome variable and measured by the BMI of reproductive-aged non-pregnant women. The BMI cut-off points recommended by the WHO have been used to measure Bangladeshi women’s overweight and obesity status^(5,10,11)^. According to the WHO, women are considered overweight when their BMI is 25·0 to 29·99 kg/m^2^, obese when their BMI is ≥ 30 kg/m^2^ and overweight/obese when their BMI is ≥ 25·0 kg/m^2(19)^. However, Bangladesh being an Asian country, this study used BMI cut-off point for Asians which is lower than the WHO criteria and categorised weight status into four groups as follows: underweight (< 18·5 kg/m^2^), healthy weight (18·5–22·9 kg/m^2^), overweight (23–27·5 kg/m^2^) and obese (>27·5 kg/m^2^)^(20)^. Finally, the primary outcome variable was formed by combining overweight and obesity for a woman with a BMI of ≥23 kg/m^2^.

### Independent variables

The following individual-level variables were finally chosen following previous studies^(10,19,21)^: women’s age (15–19, 20–29, 30–39 and 40–49 years), women’s education (no formal education, primary and secondary or higher), working status (not working and working), wealth index (poorest, poorer, middle, wealthier and wealthiest), religion (Muslim and non-Muslim), household size (1–2, 3–4 and 5+), number of children (0, 1–2 and 3+), electronic media access (no and yes) and marital status (married and widowed/divorced/separated).

Demographic and health survey data do not directly provide community-level characteristics except the place of residence (urban and rural)^(19,21)^. Therefore, other community-level factors were created by aggregating the individual-level factors inside their clusters. The distribution in each community is considered to develop community-level characteristics. This study used the mean or median as a cut-off point to create community-level factors. Current analyses also included four derived community-level variables, namely community poverty (low and high)^(21)^, community women literacy (low and high), community electronic media access (low and high)^(21)^ and community women employment (low and high). Community-level factors such as community poverty, community women literacy, community electronic media access and community women employment were derived from the individual-level factors, wealth index, women’s education, electronic media access and women’s working status, respectively. The detailed descriptions of the individual- and community-level factors are described in supplementary Table 1.

**Table 1:** Individual- and community-level factors associated with overweight/obesity in reproductive-aged (15–49 years) women in Bangladesh from univariate analysis

Variable	Total		Prevalence				*P*-value
	*n*	%	Overweight, %	Obesity, %	Overweight/obesity, %	95 % CI	
Overall	70 651	100	24·3	10·9	35·2	34·9, 35·6	
Individual-level factors							
Survey year							< 0·001
2004	10 156	14·4	13·8	4·7	18·5	17·7, 19·2	
2007	9898	14·0	16·7	6·4	23·1	22·3, 23·9	
2011	16 051	22·7	21·6	9·6	31·2	30·5, 32·0	
2014	16 284	23·0	28·2	11·7	39·9	39·1, 40·6	
2017–2018	18 264	25·9	33·2	17·2	50·4	49·7, 51·2	
Women’s age (in years)							< 0·001
15–19	6785	9·6	11·6	3·3	14·8	14·0, 15·7	
20–29	24 769	35·1	22·9	8·6	31·5	30·9, 32·1	
30–39	22 141	31·3	28·1	14·0	42·0	41·4, 42·7	
40–49	16 956	24·0	26·9	13·6	40·5	39·8, 41·3	
Women’s education							< 0·001
No education	18 801	26·6	17·9	6·6	24·5	23·9, 25·1	
Primary	21 257	30·1	23·3	9·4	32·7	32·1, 33·3	
Secondary and higher	30 593	43·3	29·4	14·9	44·3	43·7, 44·8	
Working status							0·596
Not working	48 653	68·9	23·9	11·4	35·3	34·9, 35·7	
Working	21 998	31·1	25·3	9·9	35·1	34·5, 35·8	
Wealth index							< 0·001
Poorest	12 506	17·7	13·7	4·0	17·7	17·0, 18·4	
Poorer	13 037	18·5	18·1	5·6	23·7	23·0, 24·4	
Middle	13 605	19·3	23·6	7·9	31·4	30·7, 32·2	
Wealthier	14 631	20·7	29·2	11·4	40·7	39·9, 41·5	
Wealthiest	16 872	23·9	35·2	24·2	59·5	58·7, 60·2	
Religion							< 0·001
Islam	63 291	89·6	24·4	10·9	35·3	34·9, 35·6	
Others	7360	10·4	24·0	10·9	34·9	33·9, 36·0	
Household size							< 0·001
1–2	3401	4·8	27·3	11·4	38·6	37·0, 40·3	
3–4	24 919	35·3	25·6	11·9	37·5	36·9, 38·1	
≥ 5	42 331	59·9	23·3	10·3	33·6	33·1, 34·0	
Number of living children							< 0·001
≤ 2	6344	9·0	19·5	8·6	28·0	26·9, 29·1	
3–4	35 439	50·2	25·6	11·5	37·1	36·6, 37·6	
≥ 5	28 868	40·9	23·9	10·7	34·6	34·0, 35·1	
Electronic media access							< 0·001
No	31 550	44·7	18·8	6·4	25·2	24·7, 25·7	
Yes	39 101	55·3	29·0	14·7	43·7	43·2, 44·2	
Marital status							< 0·001
Married	65 612	92·9	24·7	11·0	35·7	35·3, 36·0	
Widowed/divorced/separated	5039	7·1	19·4	9·9	29·3	28·0, 30·6	
Community-level factors							
Place of residence							< 0·001
Urban	25 375	35·9	30·9	19·1	50·0	49·4, 50·6	
Rural	45 276	64·1	22·0	7·9	29·9	29·5, 30·3	
Community poverty							< 0·001
Low	36 667	51·9	26·5	13·0	39·5	39·0, 40·0	
High	33 984	48·1	22·0	8·6	30·7	30·2, 31·1	
Community women literacy							< 0·001
Low	33 981	48·1	22·2	9·3	31·6	31·1, 32·0	
High	36 670	51·9	26·4	12·5	38·8	38·3, 39·3	
Community electronic media access							< 0·001
Low	33 245	47·1	22·1	8·4	30·4	29·9, 30·9	
High	37 406	52·9	26·3	13·1	39·4	38·9, 39·9	
Community women employment							< 0·001
Low	35 997	51·0	24·2	11·3	35·5	35·0, 36·0	
High	34 654	49·0	24·4	10·6	35·0	34·5, 35·5	

### Statistical analysis

Descriptive analyses were employed to calculate the frequencies and percentages for each of the studied variables, followed by the estimation of the prevalence of overweight and obesity by the individual- and community-level factors. The Chi-square test was used to examine the differences between the group of overweight and obesity, and the significant (*P* < 0·05) variables for the Chi-square test were considered for multilevel analysis. Multilevel logistic regression modelling was applied to explore the relationships between individual- and community-level factors with the risk of being overweight or obese. Given that the individual-level factors were nested inside the community-level factors, a two-level multilevel modelling technique was used. Associations between individual-level predictors and overweight or obesity were measured by level 1 modelling, and the community-level determinants of overweight/obesity were assessed by level 2 modelling. Four separate regression models were run in this study. Without consideration for any individual- or community-level predictor variables, Model 1 is an empty model developed to estimate the random cluster effect and measures variation among the communities (primary sampling units). Model 2 includes all selected individual-level factors in the bivariate analysis. Model 3 includes only community-level factors. The final model (Model 4) consists of individual- and community-level factors. To evaluate the best-fitted model, this study considered log-likelihood, Akaike’s information criterion and Bayesian information criterion to explain the variation of these models. A 5 % significance level and a 95 % CI were used to measure the association between selected individual- and community-level factors and overweight/obesity. Multicollinearity was checked before establishing the final models. There is no multicollinearity identified because variance influential factor is less than the threshold value, 10. Therefore, multicollinearity diagnostic results were not reported in the paper. STATA 13.0 (StataCorp, USA) was used for all statistical analyses.

## Results

Table 1 presents the distribution of overweight and obesity by individual- and community-level factors in Bangladesh. A total of 70 651 non-pregnant women were included. Among them, the highest percentage (35·1 %) was from the 20–29 years age group, 43·3 % of the women had secondary and a high level of education, and more than two-thirds of the women were not working (68·9 %). The majority (89·6 %) of the women’s religious faith were Islam. More than half of the participants had five and above household members (59·9 %) and 3–4 children (50·2 %), and 55·3 % of their families had electronic media access. At community-level factors, approximately two-thirds (64·1 %) of women were from rural areas and 52·9 % of women had high community-level electronic media access.

**Fig. 1:**
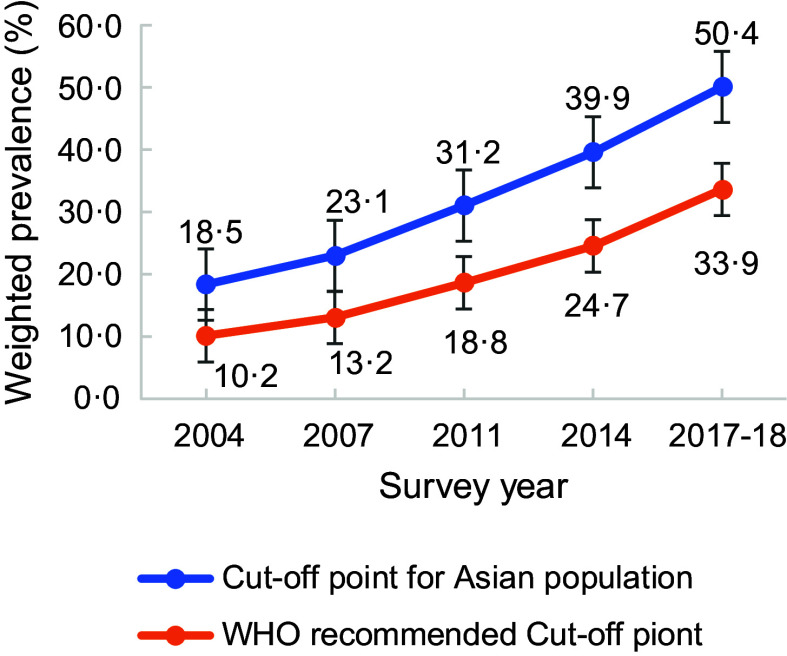
Prevalence of overweight/obesity according to WHO recommended BMI cut-off point and BMI cut-off point for the Asian population

**Fig. 2:**
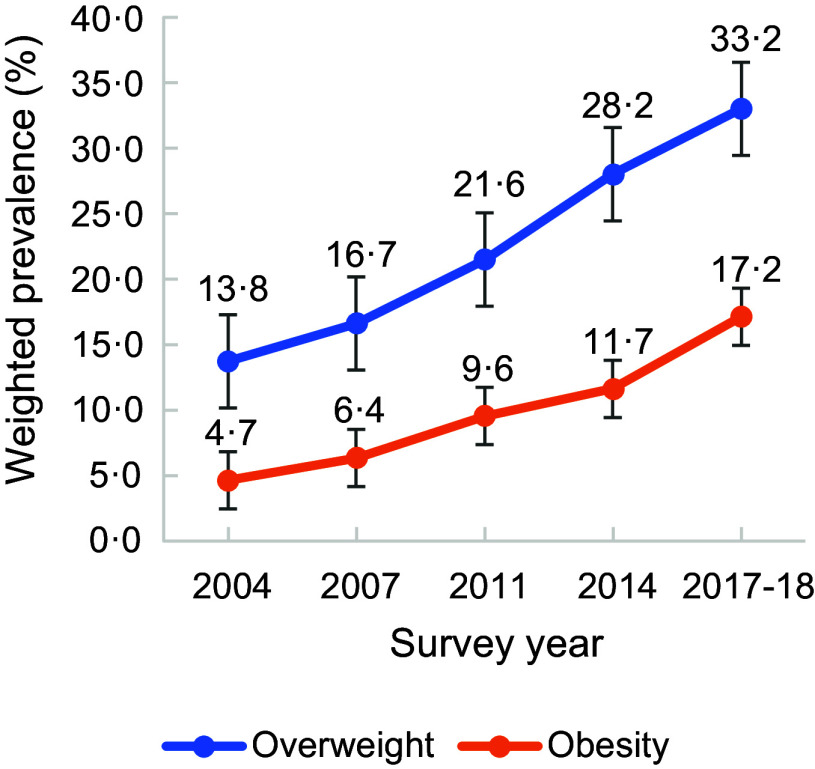
Prevalence of overweight and obesity according to BMI cut-off point for the Asian population

Figure 1 presents the prevalence of overweight/obesity according to the WHO recommended BMI cut-off point and BMI cut-off point for the Asian population. The prevalence of overweight/obesity according to the WHO suggested BMI cut-off points was lower since it followed higher cut-off points than the BMI cut-off point for the Asian population. Fig. 2 also provides the prevalence of overweight and obesity according to the BMI cut-off point for the Asian population. The prevalence of overweight and obesity has increased in recent years.

Table 1 provides the prevalence of overweight, obesity and overweight/obesity by individual- and community-level factors in Bangladesh. The overall prevalence of overweight and obesity were 24·3 % and 10·9 %, respectively. At the individual level, the prevalence of overweight/obesity has increased in recent years. The prevalence of overweight/obesity was the highest among the age group of 30–39 years (42·0 %) and lowest among the age group of 15–19 years (14·8 %). This study showed the highest rate of overweight/obesity among secondary and higher educated (44·3 %) women in Bangladesh. The prevalence of overweight/obesity increased with the wealth status of women. A small family (1–2) had a high prevalence of overweight/obesity (38·6 %). The prevalence of overweight/obesity was high among families with access to electronic media (43·7 %). Half (50·0 %) of women in the urban areas were overweight or obese at the community level. The prevalence of overweight/obesity was high among highly educated women in the community (38·8 %) and those with increased community electronic media exposure (39·4 %). All considered individual- and community-level factors except women’s working status were significantly (*P* < 0·05) associated with overweight/obesity among women.

### Multilevel analysis

Table 2 describes the results of the multivariate multilevel regression analysis for empty (Model 1), individual (Model 2), community (Model 3) and individual and community (Model 4)-level factors to measure the random effect of community and fixed effect of factors associated with overweight/obesity in non-pregnant women aged 15–49 years in Bangladesh.

**Table 2:** Individual- and community-level factors associated with overweight/obesity in reproductive-aged (15–49 years) women in Bangladesh from multivariable logistic regression analysis

Variable (Category)	Model-2			Model-3			Model-4		
	aOR	95 % CI	*P*-value	aOR	95 % CI	*P*-value	aOR	95 % CI	*P*-value
Individual-level factors									
Survey year									
2004	1·00						1·00		
2007	1·30	1·21, 1·40	< 0·001				1·28	1·19, 1·37	< 0·001
2011	1·98	1·85, 2·11	< 0·001				1·97	1·84, 2·10	< 0·001
2014	2·95	2·77, 3·15	< 0·001				2·93	2·75, 3·13	< 0·001
2017–2018	4·46	4·18, 4·76	< 0·001				4·42	4·15, 4·72	< 0·001
Women’s age (in years)									
15–19	1·00						1·00		
20–29	2·80	2·58, 3·03	< 0·001				2·93	2·57, 3·02	< 0·001
30–39	5·47	5·02, 5·96	< 0·001				5·45	5·01, 5·94	< 0·001
40–49	5·79	5·28, 6·34	< 0·001				5·79	5·28, 6·34	< 0·001
Women’s education									
No formal education	1·00						1·00		
Primary	1·30	1·24, 1·37	< 0·001				1·31	1·25, 1·38	< 0·001
Secondary or higher	1·67	1·59, 1·76	< 0·001				1·69	1·60, 1·78	< 0·001
Wealth index									
Poorest	1·00						1·00		
Poorer	1·25	1·17, 1·33	< 0·001				1·25	1·17, 1·33	< 0·001
Middle	1·70	1·60, 1·82	< 0·001				1·66	1·56, 1·77	< 0·001
Wealthier	2·38	2·23, 2·54	< 0·001				2·24	2·09, 2·39	< 0·001
Wealthiest	5·03	4·69, 5·39	< 0·001				4·41	4·10, 4·74	< 0·001
Household size									
1–2	1·00						1·00		
3–4	0·89	0·81, 0·97	0·034				0·89	0·82, 0·97	0·012
≥ 5	0·79	0·72, 0·86	< 0·001				0·80	0·73, 0·87	< 0·001
Religion									
Muslim	1·00						1·00		
Non-Muslim	0·84	0·79, 0·89	< 0·001				0·85	0·80, 0·90	< 0·001
Number of living children									
≤ 2	1·00						1·00		
3–4	1·04	0·97, 1·12	0·233				1·04	0·97, 1·12	0·252
≥ 5	0·94	0·86, 1·02	0·146				0·94	0·86, 1·02	0·150
Electronic media access									
No	1·00						1·00		
Yes	1·35	1·29, 1·41	< 0·001				1·32	1·26, 1·37	< 0·001
Marital status									
Married	1·00						1·00		
Widowed/divorced/separated	0·74	0·69, 0·80	< 0·001				0·74	0·69, 0·79	< 0·001
Community-level factors									
Residence									
Urban				1·00			1·00		
Rural				0·45	0·43, 0·46	< 0·001	0·79	0·76, 0·82	< 0·001
Community poverty									
Low				1·00			1·00		
High				0·88	0·82, 0·94	< 0·001	0·99	0·93, 1·05	0·803
Community women literacy									
Low				1·00			1·00		
High				1·25	1·18, 1·32	< 0·001	1·10	1·04, 1·15	0·001
Community electronic media access									
Low				1·00			1·00		
High				1·05	0·99, 1·12	0·089	1·02	0·96, 1·08	0·436
Community women employment									
Low				1·00			1·00		
High				1·01	0·95, 1·05	0·918	0·92	0·88, 0·97	0·002

Model 1 is an empty model.

In the final full model (Model 4), individual-level factors (such as women’s age, education, wealth index, household size, religion, electronic media access and marital status) and community-level factors (such as place of residence, community women literacy and community women employment) were significantly associated with overweight/obesity among non-pregnant women.

At the individual level, the odds of being overweight/obese were 2·93 times (adjusted odds ratio (aOR) = 2·93, 95 % CI: 2·57, 3·02) higher among those aged 20–29 years, 5·45 times (aOR = 5·45, 95 % CI: 5·01, 5·94) higher among those aged 30–39 years and 5·79 times (aOR = 5·79, 95 % CI: 5·28, 6·34) higher among those aged 40–49 years compared with women aged 15–19 years. Primary educated women (aOR = 1·31, 95 % CI: 1·25, 1·38) and secondary and higher educated women (aOR = 1·69, 95 % CI: 1·60, 1·78) were more likely to be overweight/obese compared with women who have no formal education. The odds of being overweight/obese were also significantly higher in women from the poorer (aOR = 1·25, 95 % CI: 1·17, 1·33), middle-class (aOR = 1·66, 95 % CI: 1·56, 1·77), wealthier (aOR = 2·24, 95 % CI: 2·09, 2·39) and wealthiest (aOR = 4·41, 95 % CI: 4·10, 4·74) households compared with those in women from the poorest households. Women with access to electronic media were 1·32 times (aOR = 1·32, 95 % CI: 1·26, 1·37) more likely to be overweight/obese than women with no access to electronic media. Widowed/divorced/separated women were less likely to become overweight/obese compared with married women (aOR = 0·74, 95 % CI: 0·69, 0·79). Furthermore, the odds of being overweight/obese were lower in non-Muslim (aOR = 0·85, 95 % CI: 0·80, 0·90) compared with Muslims women. However, the chance of being overweight/obese was significantly lower among women with 3–4 (aOR = 0·89, 95 % CI: 0·82, 0·97) and ≥5 (aOR = 0·80, 95 % CI: 0·73, 0·87) family members compared with their counterparts.

At the community level, the odds of being overweight/obese were lower in women from rural areas (aOR = 0·79, 95 % CI: 0·76, 0·82) and had high employment (aOR = 0·92, 95 % CI: 0·88, 0·97) than in women from an urban area and low community employment of women, respectively. The probability of being overweight and obese was increased in women from communities with high literacy (aOR = 1·10, 95 % CI: 1·04, 1·15).

Table 3 shows the results of model variation for overweight and obesity at the cluster level by multilevel logistic regression analysis. The empty model (Model 1) illustrated significant variability in the odds of overweight and obesity across communities (τ = 0·132; 95 % CI: 0·113, 0·154; *P* < 0·001). Similarly, significant variations in overweight/obesity of women existed in Model 2 (τ = 0·041; 95 % CI: 0·031, 0·052; *P* < 0·001) and Model 3 (τ = 0·062; 95 % CI: 0·050, 0·074; *P* < 0·001). After controlling the effect of individual- and community-level factors, the variance at the community level had a significant impact (τ = 0·036; 95 % CI: 0·027, 0·047; *P* < 0·001) in Model 4. The empty model (Model 1) reported an overall 3·88 % variation in the odds of overweight/obesity among women by involving the cluster difference of the characteristics (intra-cluster correlation = 3·88 %). The variability between clusters declined for the consecutive models from 3·88 % in the empty model (Model 1) to 1·23 % in the individual-level model only (Model 2), 1·84 % in the community-level model only (Model 3) and finally 1·09 % in the final model (Model 4). Proportional change in variance specified that the accumulation of forecasters to the empty model clarified an increased proportion of variation in overweight/obesity. Parallel to the intra-cluster correlation values, the proportional change in variance showed high values in the combined model (Model 4), that is, 1·09 % of the variation in overweight/obesity could be explained by the combined individual- and community-level factors. On the basis of the log-likelihood, Akaike’s information criterion and Bayesian information criterion values, Model 4 is the best-fitted model.

**Table 3:** Results from the random intercept model (a measure of variation) for overweight and obesity at cluster level by multilevel logistic regression analysis

Model summary (random effect)				
	Model 1*	Model 2†	Model 3‡	Model 4§
Communities Variance	0·132	0·041	0·062	0·036
se	0·014	0·013	0·012	0·013
95 % CI	0·113, 0·154	0·031, 0·052	0·050, 0·074	0·027, 0·047
ICC (%)	3·88 %	1·23 %	1·84 %	1·09 %
PCV (%)	Ref	68·34 %	52·65 %	71·78 %
Model fit statistics				
Log likelihood	−45821·433	−39089·874	−44733·955	−39012·142
AIC	91646·87	78223·7	89481·9	78078·28
BIC	91665·2	78425·39	89546·07	78325·75

ICC, intra-cluster correlation; PCV, proportional change in variance; AIC, Akaike’s information criterion; BIC, Bayesian information criterion.

* Model 1 is the empty model, a baseline mode l without any determinant variable.

† Model 2 is adjusted for individual-level factors.

‡ Model 3 is adjusted for community-level factors.

§ Model 4 is final model adjusted for both individual- and community-level factors.

## Discussions

This study draws required information from the five most recent BDHS data to assess individual- and community-level factors associated with overweight and obesity among non-pregnant reproductive-aged women in Bangladesh. Multivariate regression techniques were used to quantify the contribution of the individual- and community-level factors to overweight or obesity. Several studies used socio-economic and demographic characteristics, and most of the reports applied a single model such as binary logistic regression. However, the investigation of community- and individual-level factors from the Bangladesh perspective is minimal. The findings of this study revealed that approximately one-third (35·2 %) of the non-pregnant women in Bangladesh were either overweight or obese. At the individual level, the prevalence of overweight/obesity was high among women of the 30–39 years age group, wealthiest, married non-pregnant, with secondary and higher education, have 1–2 household members and with electronic media access. At the community level, the prevalence of overweight/obesity was high among non-pregnant women living in the urban area, from low community poverty, high community literacy and increased community electronic media access.

In this study, the overall pooled prevalence of overweight/obesity in the nationally representative Bangladeshi non-pregnant women was 35·2 %, and the prevalence rates of overweight and obesity were 24·3 % and 10·9 %, respectively. These values are higher than those in Nepal^(9)^ and Tanzania^(22)^. Therefore, overweight/obesity among women remains a serious concern in Bangladesh.

Several individual- and community-level factors affect the overweight/obesity of non-pregnant women. Individual-level factors (such as women’s age, education, wealth index, religion, household size, religion, number of children, electronic media access and marital status) and community-level factors (such as place of residence, community women literacy and community women employment) had a significant effect on overweight/obesity among non-pregnant women in Bangladesh. The risk of overweight/obesity increases with the women’s age. Furthermore, the high age of non-pregnant women was significantly associated with higher odds of being overweight/obese compared with those in women of the low age group (15–19 years). An earlier study conducted in Bangladesh found that older women were at a high risk of being overweight/obese^(23)^. Another study conducted in India found that overweight and obesity increased significantly with age^(24)^. The fat mass may be the main reason for being overweight/obese. The results of another study suggested that older age is associated with considerable changes in body composition because of the gradual decrease in fat-free mass and increase in fat mass after 30 years of age^(25)^. Women’s education has a positive relationship with the risk of being overweight/obese. Similar results have been reported in Bangladesh^(23,26)^ and other countries such as Tanzania^(22)^, Nepal^(9)^, Malawi^(19)^ and Ghana^(27)^. A possible reason could be that highly educated people lead a sedentary lifestyle^(26)^. Previous studies in low- and middle-income countries also revealed a significant positive relationship between education and overweight/obesity^(28)^. Even research conducted in developed countries found a negative relationship between education and excess body fat^(29)^.

Non-pregnant women from better-off households had higher odds of being overweight and obese than women from poorer households. This finding is consistent with a study conducted in low- to middle-income countries that found a significant positive association between wealth index and overweight/obesity^(30)^. A similar finding was also found in India^(24)^. The positive association between increased wealth and being overweight could be attributed to the dietary behaviour changes with high income. The intake of high energy density foods and consumption of animal and processed foods were high among the high-income households and significantly associated with overweight and obesity^(31)^. Upliftment in the wealth index has given great access to food and discharge from physical work to people in lower-income countries, leading to a high risk of being overweight and obese^(11)^. Additionally, the economically solvent and educated family often passes their time outside of the home due to job responsibilities and habituates to eating junk or fast foods outside. These activities may lead to an unhealthy life and increase the risk of overweight/obesity^(32)^. Furthermore, non-pregnant women whose families had access to electronic media were more likely to be overweight/obese than those whose families had no access to media. Television and radio are essential media sources, and women who watch television have a high risk of being overweight and obese^(27,33,34)^. Mainly television watching has been used as a proxy for sitting time. Studies that followed participants over long periods have consistently found that people who spend more time watching television are likely to be overweight and obese^(35)^.

In the context of residence, women who lived in a rural area were less likely to be overweight/obese than those who lived in an urban area. The association between place of residence and overweight and obesity is evident in past studies conducted in India^(36)^, Senegal^(37)^ and low-and middle-income countries^(22,38)^. A possible reason could be that rural women are highly engaged in physical activities such as agricultural activities, homework and other manual work as their occupation. These physical activities helped them reduce weight and hinder excessive weight gain^(39)^. Another possible reason could be that the availability of fast-food consumption, processed, packed and refrigerated foods is lower among rural women than among urban women^(22)^. Women from communities with high literacy were likely to be overweight/obese. However, high community-level literacy increases the risk of overweight and obesity^(40)^. Even health literacy is essential to understand consumer needs related to overweight and obesity^(41)^. An imminent investigation should discover the independent effects of community-level literacy on overweight and obesity among non-pregnant women in Bangladesh.

This study identified the individual- and community-level determinants of overweight and obesity among non-pregnant women aged 15–49 years in Bangladesh. The findings have important policy implications. The use of multilevel analysis helped solve practical and methodological constraints. Owing to the use of the nationally representative dataset, the ﬁndings can also be generalised among non-pregnant women.

The key strength of this study is its extensive pooled data from five successive BDHS rather than focusing on a specific survey year. Thus, the study is more diverse, and the results are more cohesive than any cross-sectional studies. This study identified important individual- and community-level factors predicting overweight and obesity among reproductive-aged women in Bangladesh. It has described community-level variables within a multilevel model framework. Therefore, unlike studies with only individual factors, it has minimised omitted variable biases. Furthermore, the results are based on five successive nationally representative surveys with large sample sizes, making the results generalisable to other similar socio-economic settings.

This study has also some limitations. The analysis was based on the secondary data, and some important variables, such as food habits, smoking, smoking habits, some non-communicable diseases risk and physical activity of women, were not included due to unavailability. The inclusion of these variables in the models might be helpful to fully understand the relationship of the selected independent variables with the overweight and obesity status of women. This study followed a cross-sectional design that cannot provide a causal relationship between explanatory variables and overweight and obesity. Further, the analysis was limited to only non-pregnant women aged 15–49 years; thus, the generalisability of the ﬁndings to all women may be limited. Despite these limitations, the results significantly contribute to the existing literature on the association of individual- and community-level factors with women’s overweight and obesity status.

## Conclusions

Individual- and community-level factors of overweight and obesity among reproductive-aged women in Bangladesh were investigated in this study. The study found that the prevalence and likelihood of being overweight/obese sharply increased over the survey period. At the individual level, women’s age, women’s education, wealth index, religion, household size, numbers of children and electronic media exposure have a significant relationship with overweight/obesity. At the community level, place of residence and community women literacy are significantly associated with a higher likelihood of being overweight/obese. Older, educated and women from the wealthiest households should be conscious about overweight/obesity and its harmful effects on health and quality of life. Media exposure should play an essential role in providing health awareness messages across all communities. Finally, executing protective interventions by government and non-government organisations may reduce the growing burden of overweight/obesity, particularly among the reproductive-aged women in Bangladesh.

## Supplementary material

For supplementary material accompanying this paper visit https://doi.org/10.1017/S1368980022001124. click here to view supplementary material
