# Supplementary material for: Multilevel exploration of individual- and community-level factors contributing to overweight and obesity among reproductive-aged women: a pooled analysis of Bangladesh Demographic and Health Survey, 2004–2018
Source: Public Health Nutr. 2022 May 16;25(8):2074–83. doi: 10.1017/S1368980022001124 (PMC9991804; doi:10.1017/S1368980022001124)
Supplement: Supplementary file 1 [file S1368980022001124sup001.docx]

**Supplementary table 1.** Definition of individual and community-level factors associated with overweight and obesity among reproductive aged women in Bangladesh.

| **Variables** | **Description** |
| --- | --- |
| **Individual level factors** |  |
| Survey year | This study considered five survey years of BDHS data and re-coded in five categories with values of 0 for 2004, 1 for 2007, 2 for 2011, 3 for 2014, and 4 for 2017-18. |
| Women’s age (in years) | Re-coded in four categories with values of 0 for 15-19, 1 for 20-29, 2 for 30-39, 3 for 40-49. |
| Women’s education | Re-coded in four categories with values of 0 for no education, 1 for primary, and 2 for secondary and higher |
| Working status | Re-coded in two categories with values of 0 for not working, and 1 for working |
| Wealth index | The datasets confined wealth index that was created using principal components analysis (PCA) coded 1 for poorest, 2 for poorer, 3 for middle, 4 for wealthier (richer), and 5 for wealthiest (richest). |
| Religion | Re-coded in two categories with a value of 0 for Islam, and 1 for others religious group (combining Hindu and the other religious categories as the women in this category are small in number). |
| Household size | The data set contained household size as a numerical variable and re-coded in three categories with values of 0 for 1-2, 1 for 3-4 and 2 for 5+. |
| Number of living children | The data set contained number of living children as a numerical variable and re-coded in three categories with values of 0 for ≤2, 1 for 3-4 and 2 for 5+. |
| Electronic media access | A composite variable created combining whether a respondent listen to radio and watch TV with a value of 0 for no access if a women lacks access to radio and TV; 1 for access to media if a woman has access any one of the media at least once a week. |
| Marital status | The data set contained marital status that was coded as 0 for married, and 1 for widowed/divorced/separated |
| **Community level factors** |  |
| Place of residence | The variable place of residence recorded as rural and urban in the dataset was retained without change. |
| Community poverty | Community poverty was created by aggregating the individual-level factors inside their clusters. The distribution of wealth index is considered to develop community poverty. This study used the mean or median as a cut-off point to create community poverty. Whether or more than mean or median of the population are in the wealth quintiles as considered high concentration of community poverty and otherwise considered low. |
| Community women literacy | Community women literacy was created by aggregating the individual-level factors inside their clusters. Primary sampling units (PSUs) is clusters were considered proxies for the community level. The distribution of women education is considered to develop community women literacy. This study used the mean or median of women education as a cut-off point to create community women literacy. Whether or more than mean or median of the population are in the women education as considered high concentration of community women literacy and otherwise considered low. |
| Community electronic media access | Aggregate values of community level electronic media access measured by the proportion of women with electronic media access derived from data on respondent’s electronic media access categorized as: bellow mean or median value of electronic media access = Low, and equal or more than mean or median value of electronic media access = High, community electronic media access. |
| Community women employment | Aggregate values of community level women employment measured by the proportion of women with working derived from data on women’s employment categorized as: bellow mean or median value of women’s working status = Low, and equal or more than the mean or median value of women’s working status = High community women employment. |
